# A *TP53* mutation model for the prediction of prognosis and therapeutic responses in head and neck squamous cell carcinoma

**DOI:** 10.1186/s12885-021-08765-w

**Published:** 2021-09-16

**Authors:** Congyu Shi, Shan Liu, Xudong Tian, Xiaoyi Wang, Pan Gao

**Affiliations:** 1 State Key Laboratory of Oral Diseases & National Clinical Research Center for Oral Diseases & Department of Head and Neck Oncology, West China Hospital of Stomatology, Sichuan University, NO.14, 3rd Section of Ren Min Nan Rd., Chengdu, 610041 Sichuan China; 2 State Key Laboratory of Oral Diseases & National Clinical Research Center for Oral Diseases & Department of General and Emergency Dentistry, West China Hospital of Stomatology, Sichuan University, Chengdu, China

**Keywords:** *TP53* mutation, Head and neck squamous cell carcinoma, Prognosis, Immunotherapy, Chemotherapy, Therapeutic response

## Abstract

**Background:**

Tumor protein *p53 (TP53*) is the most frequently mutated gene in head and neck squamous cell carcinoma (HNSC), and *TP53* mutations are associated with inhibited immune signatures and poor prognosis. We established a *TP53* mutation associated risk score model to evaluate the prognosis and therapeutic responses of patients with HNSC.

**Methods:**

Differentially expressed genes between patients with and without *TP53* mutations were determined by using data from the HNSC cohort in The Cancer Genome Atlas database. Patients with HNSC were divided into high- and low-risk groups based on a prognostic risk score that was generated from ten *TP53* mutation associated genes via the multivariate Cox regression model.

**Results:**

*TP53* was the most common mutant gene in HNSC, and *TP53* mutations were associated with immunogenic signatures, including the infiltration of immune cells and expression of immune-associated genes. Patients in the high-risk group had significantly poorer overall survival than those in the low-risk group. The high-risk group showed less response to anti-programmed cell death protein 1 (*PD-1*) therapy but high sensitivity to some chemotherapies.

**Conclusion:**

The risk score based on our *TP53* mutation model was associated with poorer survival and could act as a specific predictor for assessing prognosis and therapeutic response in patients with HNSC.

**Supplementary Information:**

The online version contains supplementary material available at 10.1186/s12885-021-08765-w.

## Introduction

Head and neck cancers mainly refer to a group of malignancies that originate from the moist mucosal surfaces lining the oral cavity, pharynx (including nasopharynx, oropharynx, and hypopharynx), larynx, and paranasal sinuses and nasal cavity. Head and neck squamous cell carcinoma (HNSC) begins in the squamous cells of mucosa and accounts for more than 90% of all head and neck cancers and is associated with high lethality [1, 2]. HNSC is estimated to cause over 600,000 new cases and 380,000 deaths annually worldwide [3, 4]. The consumption of tobacco and alcohol contributes to nearly 75% of HNSC morbidity [5]. Additionally, human papillomavirus (HPV) has been proven to be a vital risk factor for a subset of HNSC, oropharyngeal squamous cell carcinoma (OPSC) [6]. Evidence shows that HPV status is related to the prognosis of patients with HNSC. HPV^+^ HNSC, particularly HPV^+^ OPSC, is associated with a more favorable prognosis than HPV^−^ HNSC, although the effect of HPV outside the oropharynx is still unclear [7]. Consequently, the American Joint Committee on Cancer (AJCC) downgraded the stage of patients with HPV^+^ OPSC in its eighth-edition staging manual [8]. However, the optimal therapeutic methods should be determined based on more rigorous trials of definitive therapy [9]. As a result, authentication of predictive biomarkers will be beneficial to the evaluation of prognosis and surveillance of therapeutic response on HPV^+/−^ in HNSC.

Tumor protein p53 (*TP53*), encoding protein p53, is the most frequently mutated gene in HNSC [10], but the mutation is absent in the HPV^+^ subgroup [11]. Functioning as a tumor suppressor gene (TSG), *TP53* regulates multiple biological processes, including DNA repair, cell cycle arrest, apoptosis, senescence, maintenance of stem cells, and angiogenesis [12]. The loss of wildtype *TP53* or gain of oncogenic *TP53* function, predominately caused by missense mutations, is associated with inhibited immune signatures [13], poorer prognosis and resistance to radio−/chemotherapy [14], which makes the status of *TP53* to be an effective predictive biomarker to evaluate prognosis and surveillance to therapeutic responses in HNSC. Given the tumor heterogeneity in the immune microenvironment of HNSC, we hypothesized that some immune-associated signatures influenced by *TP53* mutations might play crucial roles in assessing prognosis and therapeutic responses and even guiding clinical treatment for HNSC. Thus, in our current study, we established a *TP53* mutation-related risk score model, which may affect the therapeutic response of HNSC patients.

## Material and methods

### Identification of differentially expressed genes (DEGs) and gene set enrichment analysis (GSEA)

The RNA-seq data (raw counts) from The Cancer Genome Atlas (TCGA) were normalized using the voom function from the “limma” package, and DEGs between patients with (*n* = 340) and without (*n* = 160) *TP53* mutations in the TCGA HNSC cohort were acquired by “limma” in the standard comparison mode [15]. The DEG threshold was set at |log2-fold change| ≥ 1 and *P* value adjusted < 0.05. KEGG GSEA annotation of DEGs was performed using the R package “clusterProfiler” [16–19]. The GSEA enrichment threshold was set at *P* value < 0.05, FDR q value < 0.25 and |NES| > 1.0 [20, 21]. *TP53* was re-annotated based on the results of the HNSC mutec maf file downloaded by TCGA-GDC and intersected with the tumor transcriptome. The tumor samples were divided into groups of wildtype and mutate-benign (including that the IMPACT result was LOW, as well as the IMPACT result is LOW and the PolyPhen results were benign) and mutate-non-benign according to the status of *TP53*. Their sample counts are 160 and 27 and 313 respectively.

### Construction of the *TP53*-associated prognostic model

Initially, univariate Cox analysis with *P* < 0.001 was used to evaluate the relationship between DEGs and the survival time of HNSC patients [22]. To further narrow the scope of the candidate prognostic DEGs, we adopted the multivariate Cox regression and step backward Cox regression after primary filtration [23]. The linear combination of the regression coefficient (β) derived from the multivariate Cox regression model multiplied by its mRNA level generated a prognostic risk score with ten genes. The risk score for each patient was calculated based on the risk score formula: Risk Score = expression of gene 1 × β 1 + expression of gene 2 × β 2 + … expression of gene n × β n [24]:

$$ Risk\ Score=\sum \limits_{i=1}^n\left({Expression}_i\times \beta i\right) $$

After that, the patients were divided into high- and low-risk groups by setting the median value of risk scores as cutoff value. The overall survival (OS) of these two groups was calculated by the Kaplan-Meier method with the log-rank test. Receiver operating characteristic (ROC) curves were generated to assess the predictive performance of the prognostic model [25]. The expression patterns of DEGs in this prognostic model were visualized by the “pheatmap” package (https://cran.r-project.org/web/packages/pheatmap/index.html).

### Validation of the prognostic model in external dataset

The predictive performance of the gene signature model was further validated in GSE65858. Samples with an overall survival time of less than 60 days were filtered, and then they were divided into high- or low-risk groups according to the formula of the risk score derived from the training dataset. Kaplan–Meier (KM) survival analysis and receiver operating characteristic (ROC) curves were used to evaluate the predictive power of the gene signature, and the prognostic performance of other clinicopathological factors was also analyzed. *TP53* mutational status was not available in this cohort.

### Evaluation of the immune microenvironment between the high-risk and low-risk groups

We used the CIBERSORT algorithm to analyze the fractions of 22 immune cell types using 1000 permutations and the LM22 gene signature of TCGA-HNSC samples in the R language as recommended by the authors [26]. The result was subsequently analyzed.

### Immunotherapeutic and chemotherapeutic response prediction

In recent years, immune checkpoint inhibitors have been proven to be a promising therapy for various cancers. Inhibitors targeting programmed cell death protein 1 (*PD-1*) and *CTLA-4* may enhance antitumor immunity, while tumor immune escape restricts the application of immune checkpoint blockade. The computational method of Tumor Immune Dysfunction and Exclusion (TIDE) modeled immune escape in tumors with distinct levels of cytotoxic T lymphocytes by using both T cell dysfunction and exclusion signatures [27]. In this current study, TIDE was used to predict clinical responses of HNSC to immune checkpoint inhibitors by website tools (http://tide.dfci.harvard.edu). Chemotherapy is an important method to treat HNSC, therefore the “pRRophetic” package was employed for the prediction of chemotherapeutic response, quantified by the half maximal inhibitory concentration (IC50) of each HNSC patient [28, 29]. These results were compared between the two risk groups by Kruskal-Wallis test or Chi squared test.

### Independence of the gene-related prognostic signature from other clinical features

Univariate and multivariate Cox regression analyses were conducted to determine whether the risk score calculated from the prognostic model was an independent prognostic factor for HNSC patients after considering other clinical features, including age, sex and AJCC stage, and nomograms were constructed to assess the probability of 1-, 2-, and 3-year overall survival for HNSC patients based on the signature [30].

## Results

### Identification of DEGs between HNSC patients with and without *TP53* mutations

**Fig. 1:**
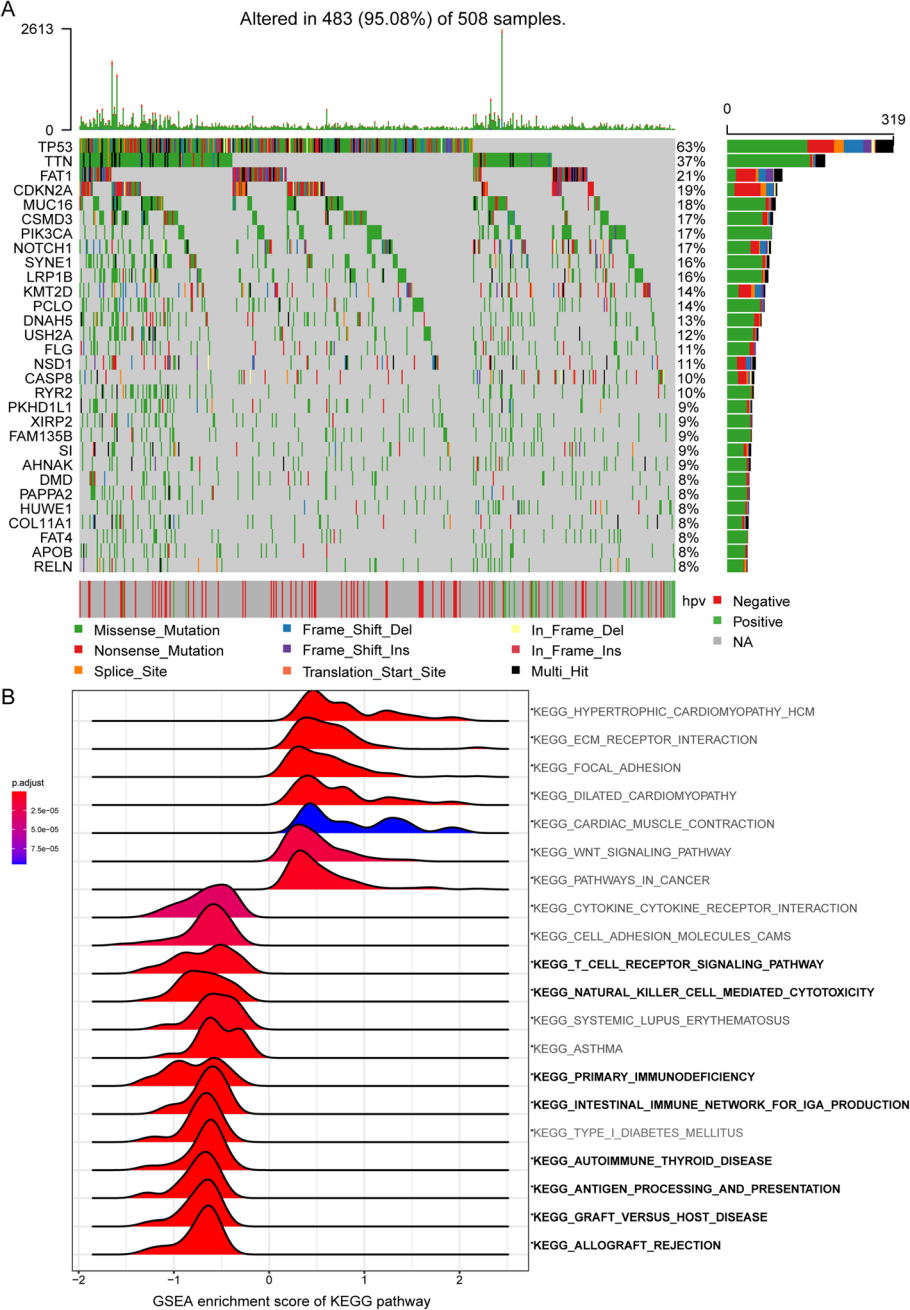
*TP53* mutation-associated genes in the TCGA HNSC cohort and GSEA of KEGG pathways. **A** The mutated genes were listed according to their mutation frequencies, considering the status of HPV infection. The mutations include missense mutations, nonsense mutations, splice-site mutations, frameshift mutations, and in-frame mutations. The oncoplot were drawn in maftools package. **B** Gene set enrichment analysis (GSEA) was performed to enrich the KEGG pathways in genes that were related to *TP53* mutation. Significant enrichments in immune-related KEGG pathways are labeled in bold. A false discovery rate (FDR) of less than 0.05 and an absolute value of the enrichment score (ES) of greater than 0.5 were defined as the cutoff criteria. The analysis were finished using clusterProfiler package

We identified mutations using data from the HNSC cohort in TCGA and found that *TP53* was the most frequently mutated gene, occurring in 63% of cases, and its mutations were mutually exclusive with HPV presence in HNSC. The difference in clinical characteristics and immune cell infiltration between HPV^−^ and HPV^+^ HNSC were analyzed. Tumor inhibition associated immune cells, including CD8 T cells, CD4 memory activated T cells, and follicular helper T cells, were more abundant in HPV^+^ HNSC. And the tumor promotion associated immune cells, including Macrophages M2, CD4 memory resting T cells, and Macrophages M0, were more abundant in HPV^−^ HNSC (Supplementary Tables 1 and 2). The majority of *TP53* mutations were missense mutations, followed by nonsense mutations and frameshift deletions (Fig. 1A). In this study, we divided patients with HNSC into *TP53*-mutated and wildtype groups and investigated the corresponding DEGs. In total, 508 upregulated genes and 197 downregulated genes were discerned. To elucidate the functions of DEGs, GSEA was carried out. As shown in Fig. 1B, immune activated signals, including the T cell receptor signaling pathway, natural killer cell mediated cytotoxicity, primary immunodeficiency, and antigen processing and presentation, were enriched in *TP53* wildtype HNSC tumor tissues, which indicated that the *TP53* mutation status may influence the immune response to HNSC. In the re-analysis, 35 mutate-benign were eliminated for differential expression analysis. The results showed that the 10 genes in the risk score model were still in the significant DEGs (Supplementary Figure 1A), which were shown in the volcano map. We supplemented the Kaplan-Meier analysis according to the annotation results. There was no significant difference in overall survival between mutate-benign group and mutate-non-benign group (*P* > 0.05; Supplementary Figure 1B). Compared with wildtype group, the group of mutate-non-benign had poorer prognosis (*P* < 0.05; Supplementary Figure 1B). The overall survival between groups divided by the result of PolyPhen annotation was not statistically significant (Supplementary Figure 1C).

### Construction of the prognostic signature model based on DEGs

**Fig. 2:**
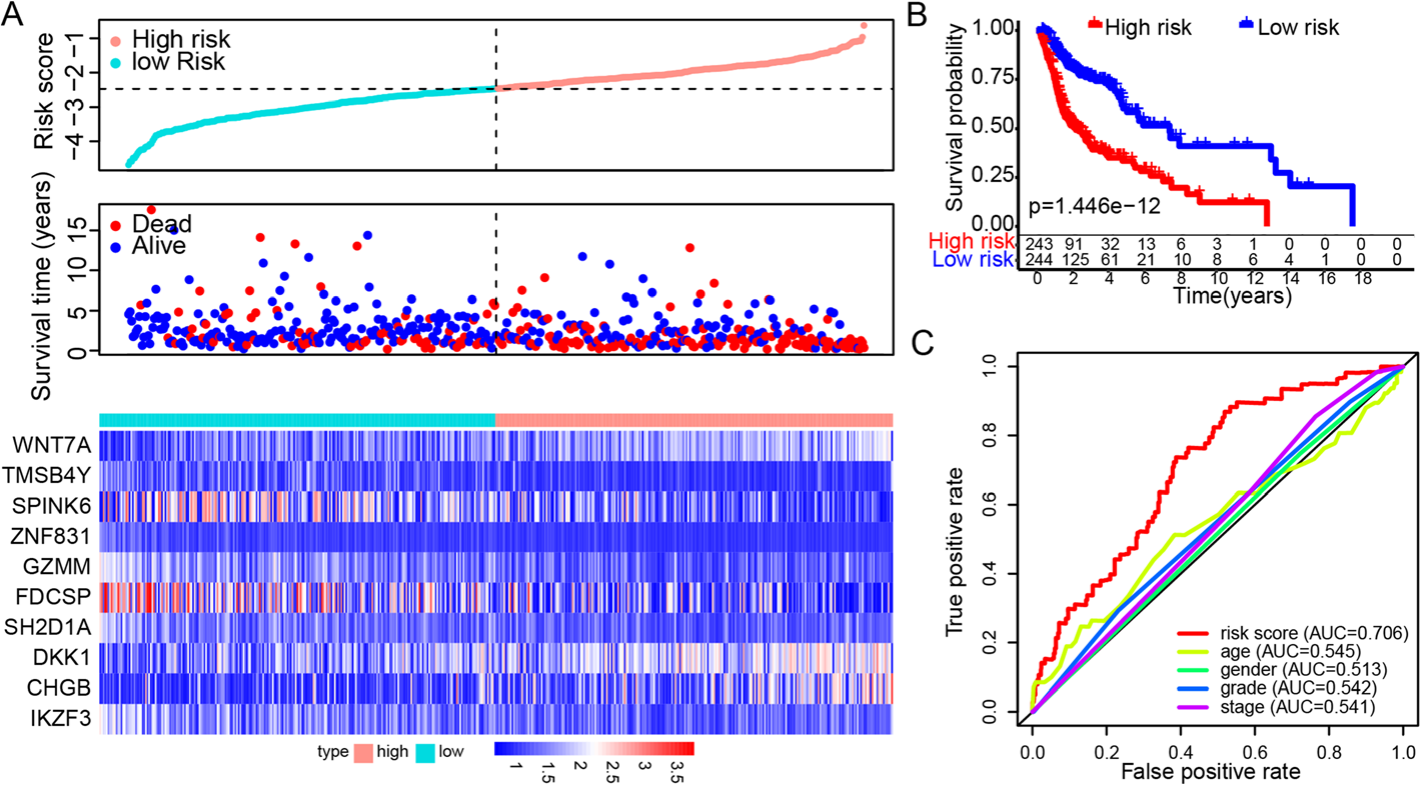
Construction of the DEG-based gene signature prognostic model. **A** Risk score, vital status and heatmap of prognostic genes in the high- and low-risk groups. **B** Kaplan-Meier survival curves of the relative overall survival of high- and low-risk patients. **C** The ROC curve for one-year survival of the gene signature and clinical features. DEGs were identified between *TP53* mutated and wildtype TCGA-HNSC samples (Padj < 0.05, |logFC| > 1). Prognostic DEGs were screened with coxph *P* value < 0.001, these genes were displayed in Supplementary Table 3. The risk model was constructed after stepwise coxph analysis with survival package. The information of ten genes in model were summarized in supplementary Table 4

Thirty genes with *P* < 0.001 were left after the univariate Cox analysis of DEGs (508 upregulated DEGs + 197 downregulated DEGs). More information of the 30 genes were displayed in Supplementary Table S3. Then we adopted backward stepwise multivariate Cox regression analysis of the 30 genes, ten genes were identified to build the prognostic model. A risk score based on ten genes for predicting prognostic value was calculated based on the formula: RiskScore = *WNT7A* * 0.275 + *TMSB4Y* * (− 0.541) + *SPINK6* * (− 0.196) + *ZNF831* * (− 3.2) + *GZMM* * (− 0.417) + *FDCSP* * (− 0.058) + *SH2D1A* * 0.584 + *DKK1* * 0.102 + *CHGB* * 0.131 + *IKZF3* * 0.629. The basic information of the ten genes was shown in Supplementary Table 4. Most of the ten genes are associated with cancer and immune signaling pathways. *WNT7A*, *SPINK6*, *DKK1*, and *CHGB* were upregulated, and the rest of the ten genes were downregulated in *TP53*-mutated group in comparison with *TP53*-wildtype group (Supplementary Figure 2). The vital status and heatmap of prognostic genes are presented (Fig. 2A). Kaplan-Meier survival curves showed that patients classified in the high-risk group were more likely to have significantly worse overall survival than those classified in the low-risk group (*P* < 0.0001; Fig. 2B). The ROC curves and corresponding area under the curve (AUC) showed that the model based on the risk score was better than the model based on clinical characteristics, including age, sex, grade and stage (Fig. 2C).

### External validation of the prognostic signature model based on DEGs

**Fig. 3:**
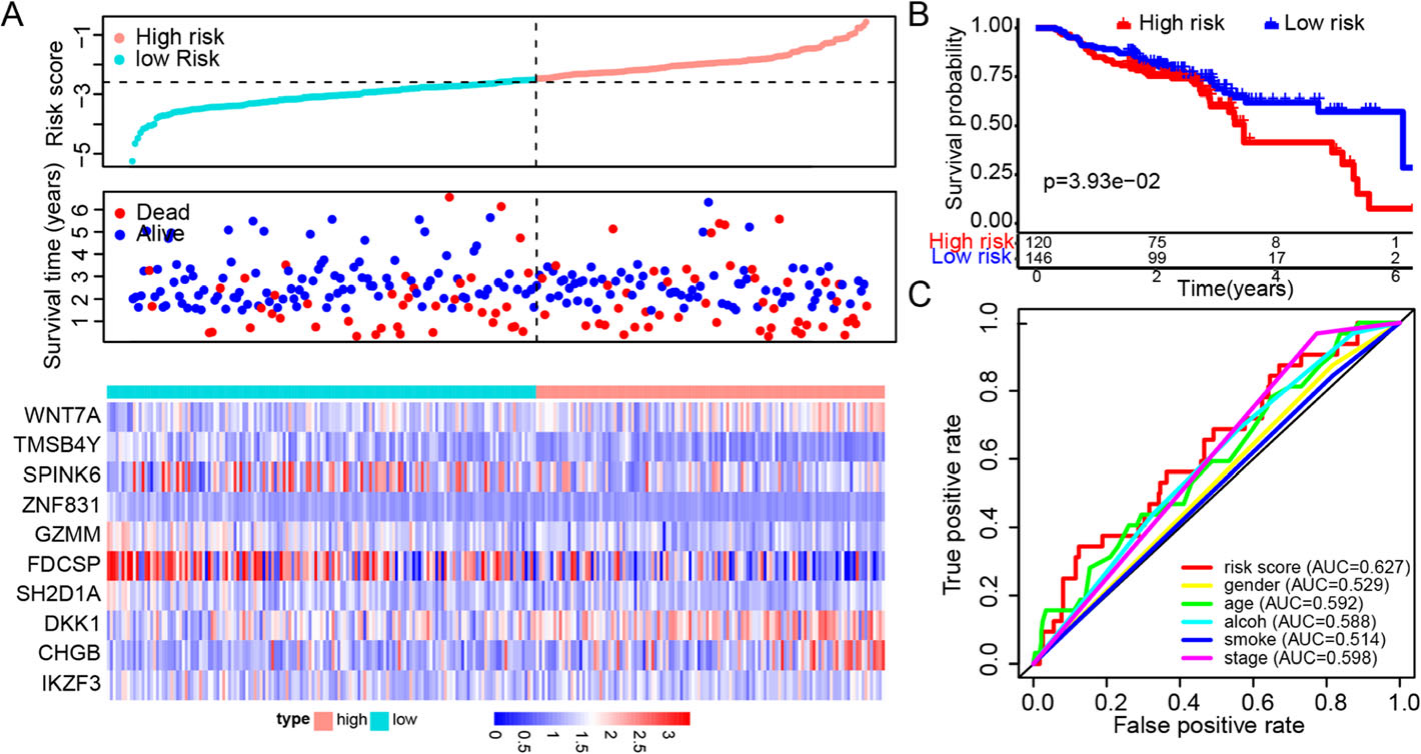
Validation of the DEG-based gene signature prognostic model in GSE65858. **A** Risk score, vital status and heatmap of prognostic genes in the high- and low-risk groups. **B** Kaplan-Meier survival curves of the relative overall survival of high- and low-risk patients. **C** The ROC curve for one-year survival of the gene signature and clinical features

The GSE65858 cohort of HNSC, a dataset from the Gene Expression Omnibus (GEO), was analyzed using the constructed model. The risk score based on ten genes for evaluating prognostic value was calculated, and the vital status and heatmap of prognostic genes were indicated (Fig. 3A). Kaplan-Meier survival analysis showed that patients grouped into the high-risk set tended to have poorer overall survival than those in the low-risk group (*P* < 0.05; Fig. 3B). The ROC curves and corresponding AUC showed that the model based on risk score was better than clinic features, including age, sex, grade and stage (Fig. 3C).

### Kaplan-Meier analyses of overall survival according to *TP53* mutation status

**Fig. 4:**
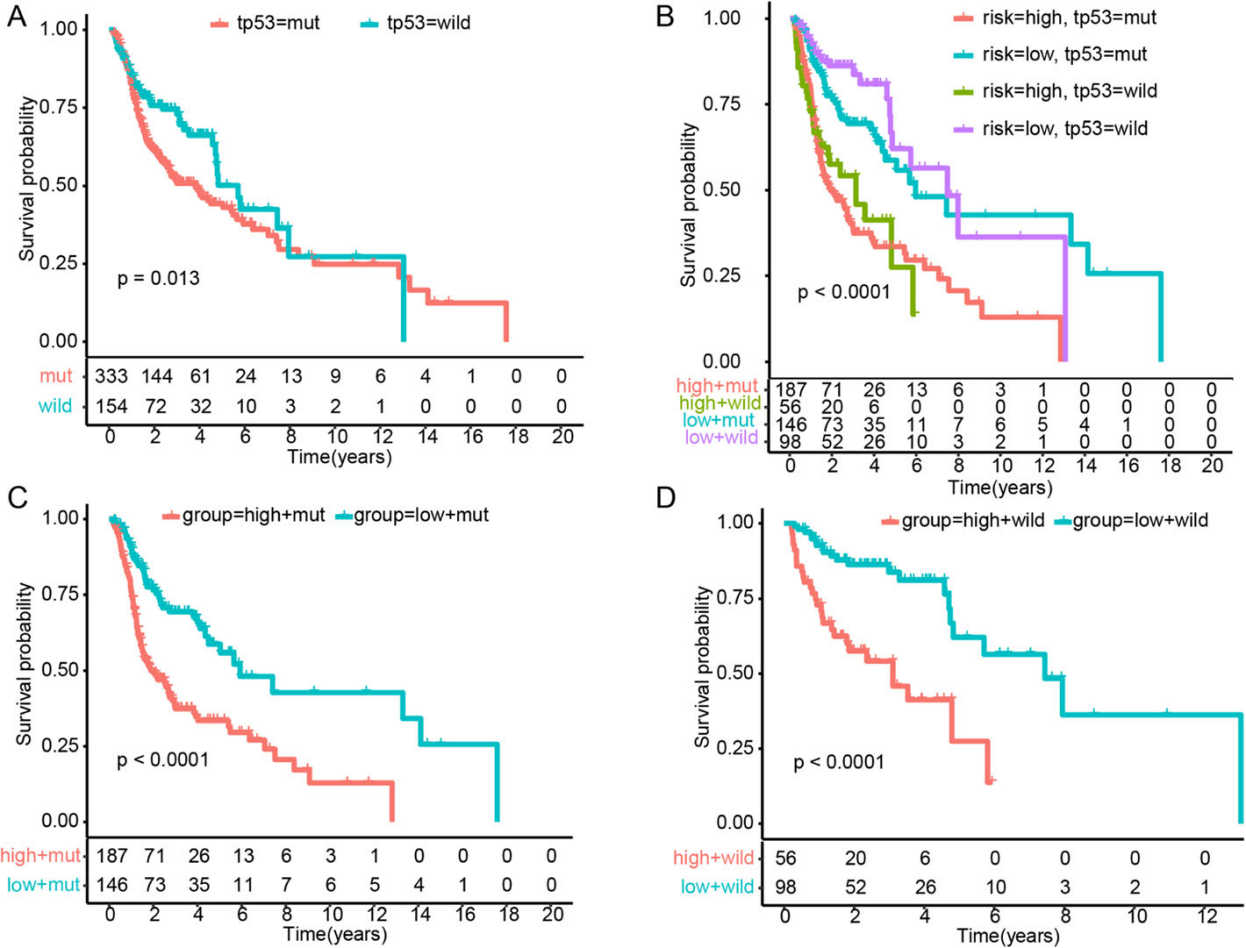
Kaplan-Meier analysis of overall survival based on the combination of *TP53* mutation status and risk score of the prognostic model. **A** *TP53* mutation vs wildtype group. **B** *TP53* mutation status with high−/low-risk group. **C** *TP53* mutation subgroup with high−/low-risk group. **D** *TP53* wildtype subgroup with high−/low-risk group

Consistent with a previous report [31], our study found that *TP53* mutation status was associated with prognosis; namely, HNSC patients with *TP53* mutation had poorer prognosis (*P* < 0.05; Fig. 4A). To elucidate whether the risk score is independent of *TP53* mutation, patients with HNSC were grouped into distinct sets according to *TP53* mutation or wildtype with high- or low-risk status (Fig. 4B). The patients with both a high-risk score and *TP53* mutations were inclined to have the worst overall survival in comparison with any other single or combined factors (*P* < 0.0001; Fig. 4B). However, the status of HPV was not associated with the prognosis of HNSC (Supplementary Figure 3A). The risk score was also independent of the HPV status (Supplementary Figure 3B-D). Kaplan-Meier overall survival curves of the two sets based on the risk score were significantly distinct in the mutation and wildtype cohorts of HNSC, which indicated that the risk score was an independent predictor of prognosis (*P* < 0.0001; Fig. 4C and D).

### Immune cell infiltration landscapes of high- and low-risk patients with HNSC

**Fig. 5:**
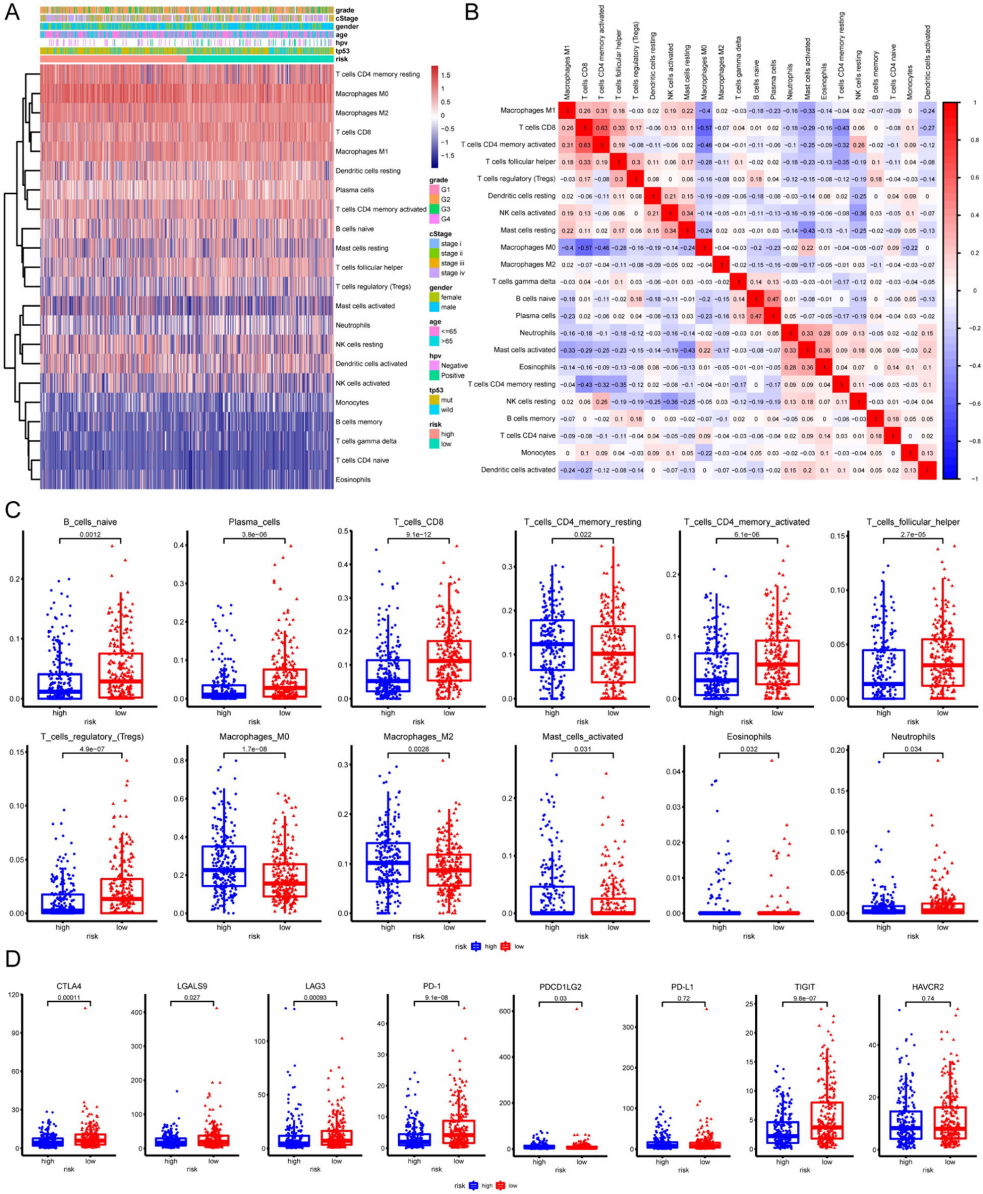
Immune cell infiltration landscapes in high- and low-risk patients with HNSC in TCGA-HNSC cohort. **A** Scaled immune cell infiltration proportions in high- and low-risk patients. The proportions were estimated by CIBERSORT through mRNA expression matrix (scaled by log2(TPM + 1)); **B** Correlation matrix for immune cells. **C** Differences of immune cell infiltrations between high- and low-risk patients. **D** Differences of immune-associated genes between high- and low-risk patients

Immune infiltration of patients with HNSC between high- and low-risk groups was then elucidated. The ratios of 22 tumor-associated immune cell types were significantly distinct between high- and low-risk patients with HNSC (Fig. 5A). The correlation matrix indicated moderate correlations between immune cell rates (Fig. 5B). High-risk patients with HNSC tended to have higher rates of CD4^+^ resting memory T cells and M0/M2 macrophages and lower rates of naive B cells, plasma cells, CD8^+^ T cells, activated memory CD4^+^ T cells, follicular helper T cells, regulatory T cells (*P* < 0.05; Fig. 5C). The difference of cells CD4^+^ resting memory T cells and M0/M2 macrophages CD8^+^ T cells, activated memory CD4^+^ T cells, follicular helper T cells between risk groups reappeared in GSE65858 cohort (*P* < 0.05; Supplementary Figure 5) More details considering HPV status were recorded in Supplementary Table 2.

Antibodies that target immune checkpoints, such as CTLA-4 or PD-1, are the current strategies for cancer immunotherapy [32]. Many studies have indicated that *LAG3*, *LGALS9*, *HAVCR2* and *TIGIT* might be next-generation immune checkpoints [33–36]. We found that high-risk patients with HNSC expressed remarkably lower levels of *CTLA4* (*P* < 0.01), *LGALS9* (*P* < 0.05), *LAG3* (*P* < 0.001), *PD-1* (*P* < 0.0001), and *TIGIT* (*P* < 0.0001), but higher levels of *PD-1* (*P* < 0.05; Fig. 5D). These results suggested that immunotherapy targeting immune checkpoints might be more effective for low-risk patients.

### Immunotherapeutic and chemotherapeutic responses of high- and low-risk patients with HNSC

**Fig. 6:**
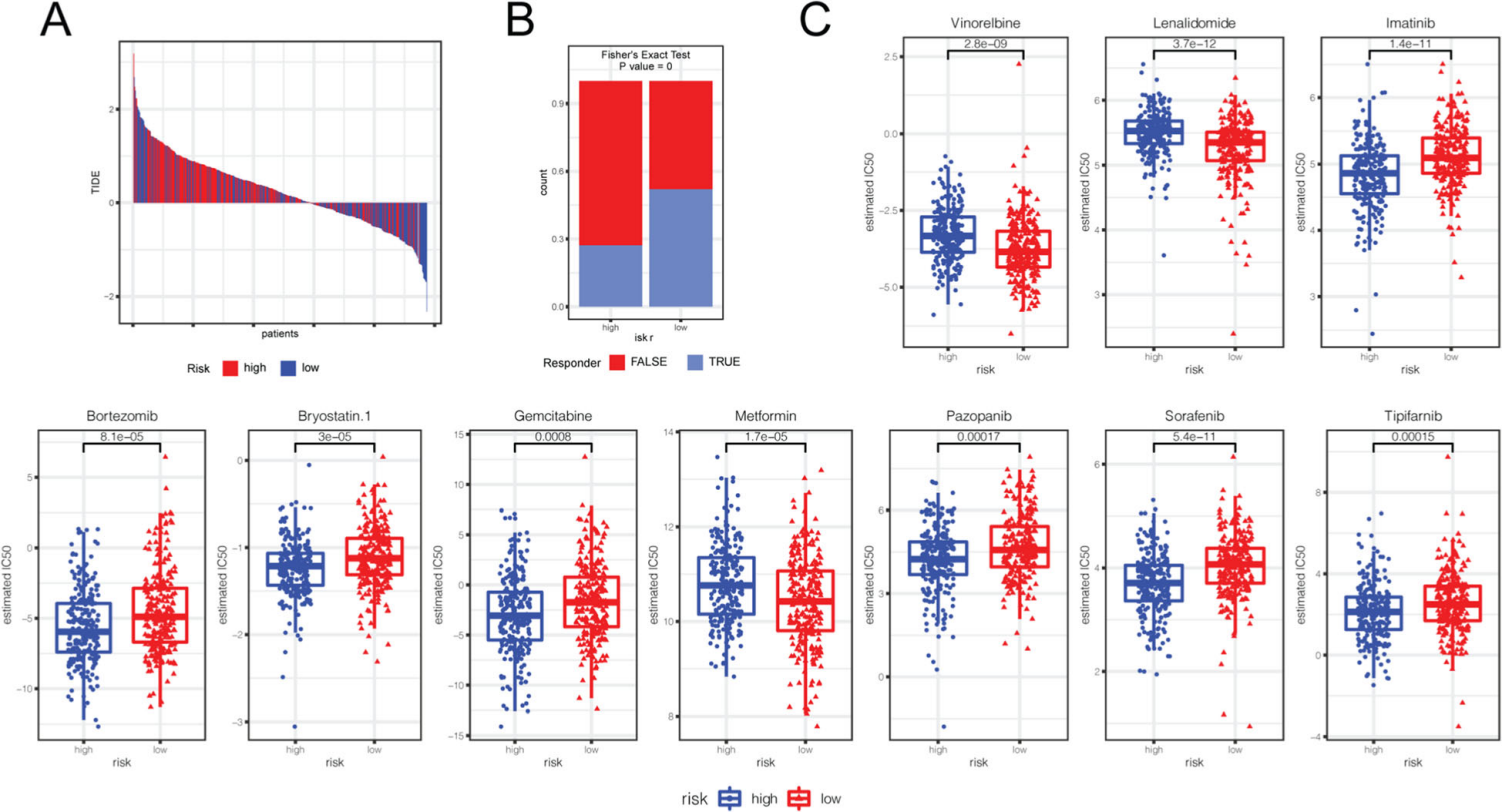
Immunotherapeutic and chemotherapeutic responses in high- and low-risk patients with HNSC. **A** Immunotherapeutic responses to anti-PD-1 therapy in high- and low-risk patients. **B** The P value is shown using Fisher’s exact test to detect whether the immunotherapeutic response rates of the high-risk and low-risk groups were significantly different. **C** Drugs in clinics or in test for HNSC with significant differential chemotherapeutic responses (*P* < 0.001) in high- and low-risk patients predicted by the pRRopheic package in the R language

Antibody targeting of immune checkpoint blockade provides a promising therapy for a variety of human cancers; however, partial and complete therapeutic resistance is commonly present in most patients. Therefore, we analyzed the clinical response to *PD-1* and a group of chemical drugs in high- and low-risk patients with HNSC. Notably, anti-PD-1 therapy was less effective in most of the high-risk patients with HNSC (*P* < 0.01; Fig. 6A and B), which is consistent with the above hypothesis. The chemotherapeutic and targeted drugs used in the clinical practice, and clinical trials in HNSC showed differences in the predicted IC50 between high- and low-risk patients with HNSC. Imatinib, Bortezomib, Bryostatin.1, Gemcitabine, Pazopanib, Sorafenib, and Tipifarnib significantly decreased the IC50 and increased the sensitivity for high-risk patients with HNSC (*P* < 0.001, Fig. 6C). Docetaxel, Erlotinib, Axitinib, Doxorubicin, Paclitaxel, and Rapamycin are also sensitive for high-risk patients with HNSC (*P* < 0.05, Supplementary Figure 4). These results suggested that most chemotherapies might be more effective for high-risk patients.

### Correlations between the prognostic signature model and clinical characteristics

**Fig. 7:**
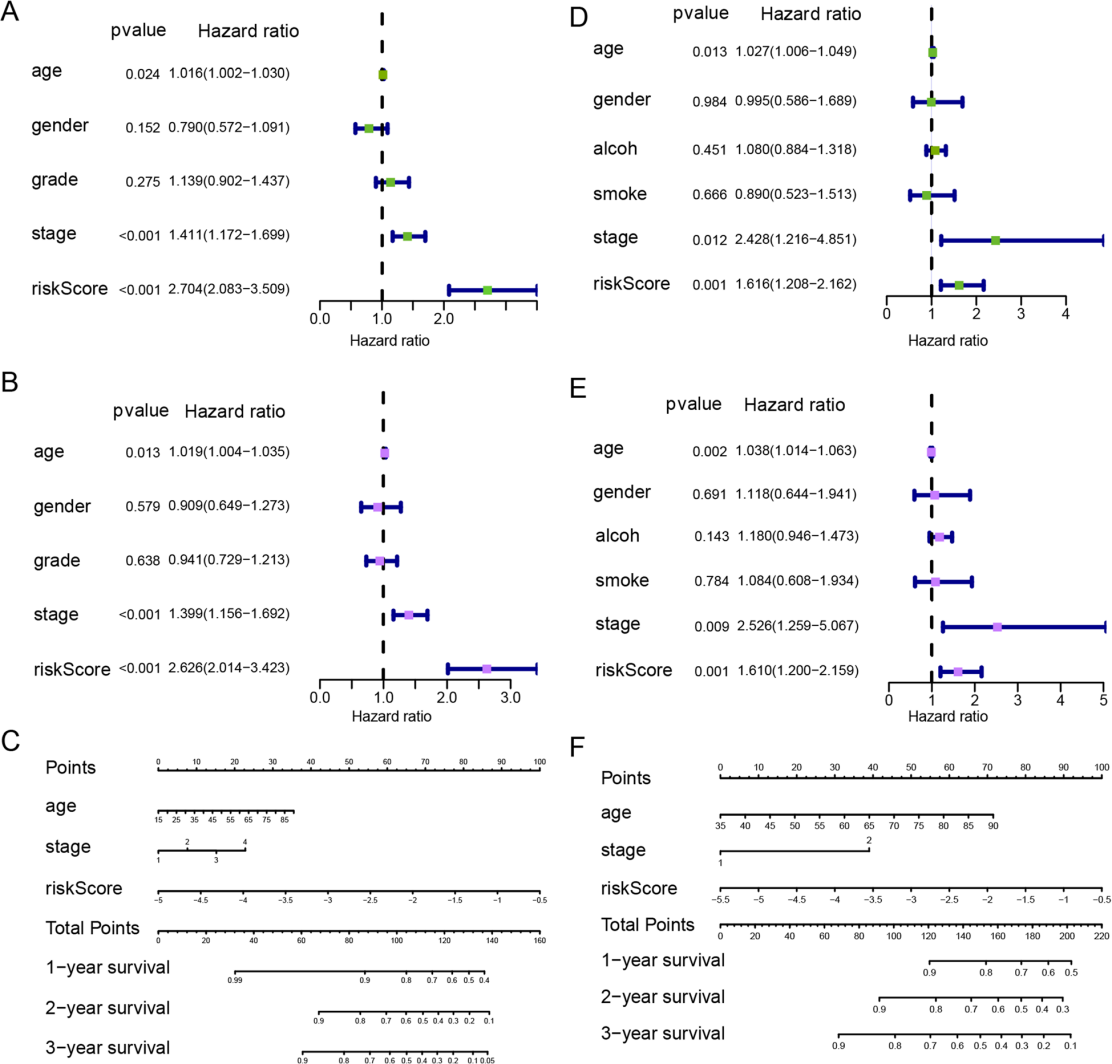
Correlation between the gene signature and clinical characteristics in the TCGA HNSC cohort and GSE65858 dataset. Univariate (**A**) and multivariate (**B**) Cox regression analyses of correlations between the gene signature and clinical characteristics with overall survival in the TCGA HNSC cohort. **C** Nomogram for predicting the 1-, 2-, and 3-year overall survival of patients with HNSC. Univariate (**D**) and multivariate (**E**) Cox regression analyses of correlations between the gene signature and clinical characteristics with overall survival in the GSE65858 dataset. **F** Nomogram for predicting the 1-, 2-, and 3-year overall survival of patients in the GSE65858 dataset

To identify whether the risk score is an independent predictive factor in comparison with traditional clinical characteristics, univariate and multivariate Cox regression analyses were performed. In the HNSC cohort of TCGA, clinical stage and risk score were associated with poorer survival and could serve as specific predictors for prognostic assessment (*P* < 0.001, Fig. 7A and B), as well as in the GSE65858 dataset (*P* < 0.01, Fig. 7D and E). A prognostic nomogram based on multivariate analysis was used to further evaluate the prognostic effect. Compared with age and clinical stage, the risk score performed better at predicting the 1-, 2-, and 3-year overall survival (Fig. 7C and F).

## Discussion

HNSC is the most common subtype of head and neck malignancies, with more than 40% mortality worldwide [37] and approximately 41% concordance with *TP53* mutation [11]. Traditionally, clinicians plan treatment of HNSC based on clinical and pathological stage, status of HPV infection (particularly in OPSC), presence of a metastatic lymph node with or without extranodal extension (ENE), and depth of invasion (DOI) in oral cavity cancer [8]. In addition, the expression of molecular biomarkers can predict prognosis and monitor responses to therapies and even be therapeutic targets, such as epidermal growth factor (*EGFR*) monoclonal antibody (cetuximab) and immune checkpoint inhibitors targeting *PD-1* [38]. Thus, the identification of novel targets or mutations is helpful for clinical strategies. An increasing number of studies have shown that the *TP53* mutation is associated with HNSC prognosis [31] and acts as a predictor of the clinical response to HNSC treatment [39]. In our current study, we identified that *TP53* was the most frequently mutated gene in 63% of HPV^−^ HNSC, the majority of which were missense mutations.

An increasing number of studies elsewhere have shown that *TP53* plays a crucial role in tumor recognition by the immune system and antitumor immunosurveillance [40–43]. However, the bilateral functions of inhibiting [42, 44] or promoting [45, 46] antitumor immune activity indicate that the tumor immunity induced by *TP53* mutations is attributed to tumor heterogeneity in different human cancers. According to the DEGs between the *TP53*-mutated and wildtype groups, GSEA analysis was performed, and we found that *TP53* mutations were associated with immune-related signaling pathways in patients with HNSC, including the T cell receptor signaling pathway, natural killer cell-mediated cytotoxicity, primary immunodeficiency, and antigen processing and presentation.

Given that the status of *TP53* mutation functions as an effective prognostic predictor and therapeutic surveillance in HNSC [31], the risk score, a novel model based on ten genes affected by *TP53*, was first established to efficiently evaluate the overall survival likelihood of patients with HNSC. The results indicated that the risk score was more effective than clinical features, including age, sex, grade and clinical stage. The discovery cohort and the replication cohort are based on two different technologies (RNA-Seq vs Bead-array) which renders the comparison hazardous and could explain why the model does not perform so well in the validation cohort. Patients grouped as high-risk were more likely to have poorer overall survival. The risk score with *TP53* mutation exerted a more significant effect for the prediction of overall survival.

Immune cell infiltration is a significant characteristic of the tumor microenvironment (TME). The dual role of immunity between tumors and the host evolves the hypothesis of cancer immunosurveillance into cancer immunoediting, which is a dynamic process consisting of three phases: elimination, equilibrium, and escape [47]. The interferon gamma (INF) produced by infiltrated lymphocytes, such as natural killer cells and natural killer T cells, induces tumor death and secretion of several chemokines, which regulate the recruitment of more immune cells, such as how cytotoxic CD8+ T cells migrate to the tumor site and kill tumor cells with an immunogenic phenotype [48]. Tumor cell variants with genetic mutations or epigenetic changes acquire a nonimmunogenic phenotype and resistance to the immune system and then survive the elimination phase and enter the escape phase [47].

A growing amount of evidence shows that the presence of tumor-infiltrating lymphocytes (TILs) is associated with a favorable prognosis and a better response to therapies in a wide range of malignancies, including epithelial ovarian cancer, esophageal carcinoma, gastric carcinoma, colorectal cancer, lymph node melanoma, and primary cutaneous melanoma [49–54]. The prognostic role of TILs in HNSC is highly consistent with previous studies on other cancers. An immunohistochemical investigation showed that high CD3^+^ or CD8^+^ TILs in oral cancer were related to an improved prognosis [55], which was confirmed by a systematic review and meta-analysis [56]. A large retrospective study on HNSC also indicated that CD4^+^ and CD8^+^ T cells were independent factors for better overall survival and recurrence-free survival [57].

Loss of function or mutation of *TP53* was perceived to be associated with inhibition or escape from antitumor immune responses [58]. Li L et al. reported that immune-stimulatory and immune-inhibitory signatures, CD8+/CD4+ regulatory T cells, pro−/anti-inflammatory cytokines, and M1/M2 macrophages were lower in *TP53*-mutated HNSC than in the wildtype subgroup [59]. Recently Huo M et al. established a prognostic risk model based on the TME score and found that patients in the high-risk group were more likely to have worse prognosis and a high frequency of *TP53* mutation and expressed lower immune activation genes, *CXCL9* and *CXCL10*, and immune checkpoint genes, *IDO1* and *HAVCR2* [60]. Consistently, our current study found that high-risk patients with HNSC generally had lower proportions of naive B cells, plasma cells, CD8+ T cells, activated memory CD4+ T cells, follicular helper T cells, and regulatory T cells. HNSC patients in the high-risk group expressed significantly lower levels of immune checkpoint genes, including *PD-1*, *CTLA4*, *LGALS9*, *LAG3*, and *TIGIT*.

*PD-1* is an immune checkpoint that normally inhibits T cell activation, and tumor cells can suppress the antitumor immune response by upregulating the expression of PD-1 ligands [61]. Given that *PD-L1* is highly expressed in HNSC [62], inhibitors of *PD-1* or *PD-L1* are thought to be a promising strategy to enhance T cell-mediated antitumor immunity and improve patient survival [63, 64]. In our current study, HNSC patients in the high-risk group tended to be unresponsive to anti-PD-1 therapy. However, high-risk patients with HNSC were more sensitive to 28 chemotherapies. Collectively, the reason why high-risk patients with HNSC have poorer prognosis may be attributed to higher immunosuppression or lower immunoreactivity in the TME, which promotes tumor growth, metastasis, and resistance to therapies. These data suggested that the risk score system might be beneficial to individualized treatment for patients with HNSC.

Clinical stage and risk score significantly affected the overall survival of patients with HNSC, when compared with sex, grade, and alcohol and smoke consumption. Furthermore, the ten-gene risk score signature was associated with poorer survival and functioned as a predictor for prognostic assessment.

## Conclusion

Our study suggested that the gene signature combined with the status of *TP53* mutation offered a more effective method to evaluate prognosis and response to immunotherapies and chemotherapies for patients with HNSC.

## Supplementary Information

**Additional file 1: Supplementary Figure 1.** DEGs and KM survival plot of patients with non-benign *TP53* mutation and wildtype. (A) The volcano plot of DEGs between non-benign *TP53* mutation and wildtype patient tissues. The DEG analysis were finished by using limma package. (B) The survival plot of HNSC patients classified by reannotated *TP53* mutation status according SnpEFF IMPACT and PolyPhen annotation results, including benign (IMPACT was LOW and PolyPhen was benign), non-benign (other mutations: IMPACT was MODERATE/HIGH or PolyPhen was possibly_damaging/probably_damaging). (C) The survival plot of HNSC patients classified by PolyPhen annotated *TP53* mutation status. The DEGs were identified by limma package (padj < 0.05, |logFC| > 1).

**Additional file 2: Supplementary Figure 2.** The boxplot of ten prognostic genes expression in model of TCGA HNSC samples of *TP53* mutation and wildtype. The *p* values were calculated by Kruskal-Wallis test.

**Additional file 3: Supplementary Figure 3.** The KM survival plot TCGA HNSC patients stratified by HPV status and risk model. (A) HPV^+^ vs HPV^−^ group. (B) HPV^+^ with high−/low-risk group. (C) HPV^+^ subgroup with high−/low-risk group. (D) HPV^−^ subgroup with high−/low-risk group.

**Additional file 4: Supplementary Figure 4.** Drugs in clinics or in test for HNSC without significant differential chemotherapeutic responses (*p* ≥ 0.001) in high- and low-risk patients. The results were predicted by the pRRopheic package in the R language, and the difference of IC50 were assessed with Kruskal-Wallis test.

**Additional file 5: Supplementary Figure 5.** Immune cell infiltration landscapes in high- and low-risk patients with HNSC in GSE65858 cohort. (A) Correlation matrix for immune cells.; (B) Scaled immune cell infiltration proportions in high- and low-risk patients (C) Differences of immune cell infiltrations between high- and low-risk patients. The difference of cell fractions were assessed with Kruskal-Wallis test.

**Additional file 6: Supplemental Table 1.** The immune and clinic characteristics between HPV negative patients and positive patients in TCGA HNSC. Briefly speaking HPV + HNSC has more infiltrated immune cells, especially tumor suppressive cells like memory B cell, CD8 T cells, activated memory CD4 T cells, T cells follicular helper, and less pro-tumor cells like Macrophages M2. Interestingly HPV + HNSC has a greater proportion of low risk group, and those HPV positive patients were younger (median age:57 vs 61) and of high pathological grade.

**Additional file 7: Supplementary Table 2.** The immune cell infiltration differences between patients of high risk and low risk stratified by HPV status. The immune cell proportion differences were mainly present in HPV-HNSC patient. Significant *p* values were marked boldly.

**Additional file 8: Supplementary Table 3.** Univariate Cox analysis results of the 30 significant genes. Ten genes in risk model were marked boldly. Padj value were calculated by Bonferroni correction.

**Additional file 9: Supplementary Table 4.** Information of the ten genes in the prognostic model. Most of the ten genes were correlated with tumor or immune functions. Padj value and HR from coxph analysis and coefficients from risk model formula were displayed in the table.

## Data Availability

We downloaded VarScan 2-based somatic mutation data, RNA sequencing and clinical information data of the TCGA HNSC cohort from UCSC-Xena, dated August 28th, 2019. As a result, a total of 500 HNSC samples were enrolled in this analysis. The GSE65858 microarray dataset (https://www.ncbi.nlm.nih.gov/geo/query/acc.cgi?acc=GSE65858) comprises 270 HNSC specimens with gene expression profiles and their associated clinical characteristics [65]. All data were normalized in the R computing environment using the limma package. All analyses were conducted in accordance with relevant regulations and guidelines.
