# Supplementary figures and images for: A TP53 mutation model for the prediction of prognosis and therapeutic responses in head and neck squamous cell carcinoma
Source: BMC Cancer. 2021 Sep 16;21:1035. doi: 10.1186/s12885-021-08765-w (PMC8447564; doi:10.1186/s12885-021-08765-w)

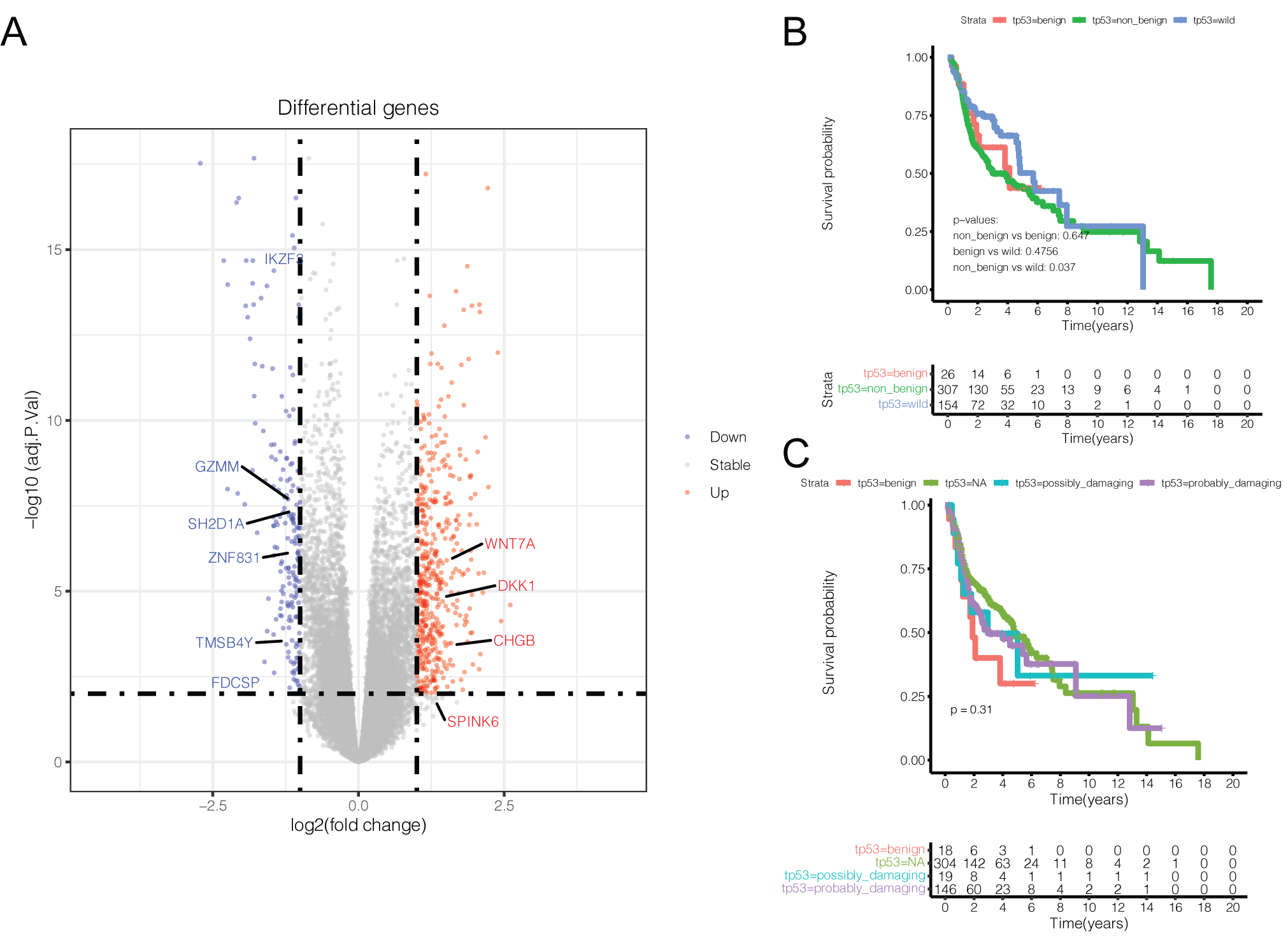

Supplement: Supplementary file 1 — Additional file 1: Supplementary Figure 1. DEGs and KM survival plot of patients with non-benign TP53 mutation and wildtype. (A) The volcano plot of DEGs between non-benign TP53 mutation and wildtype patient tissues. The DEG analysis were finished by using limma package. (B) The survival plot of HNSC patients classified by reannotated TP53 mutation status according SnpEFF IMPACT and PolyPhen annotation results, including benign (IMPACT was LOW and PolyPhen was benign), non-benign (other mutations: IMPACT was MODERATE/HIGH or PolyPhen was possibly_damaging/probably_damaging). (C) The survival plot of HNSC patients classified by PolyPhen annotated TP53 mutation status. The DEGs were identified by limma package (padj < 0.05, |logFC| > 1). [file 12885_2021_8765_MOESM1_ESM.tif]

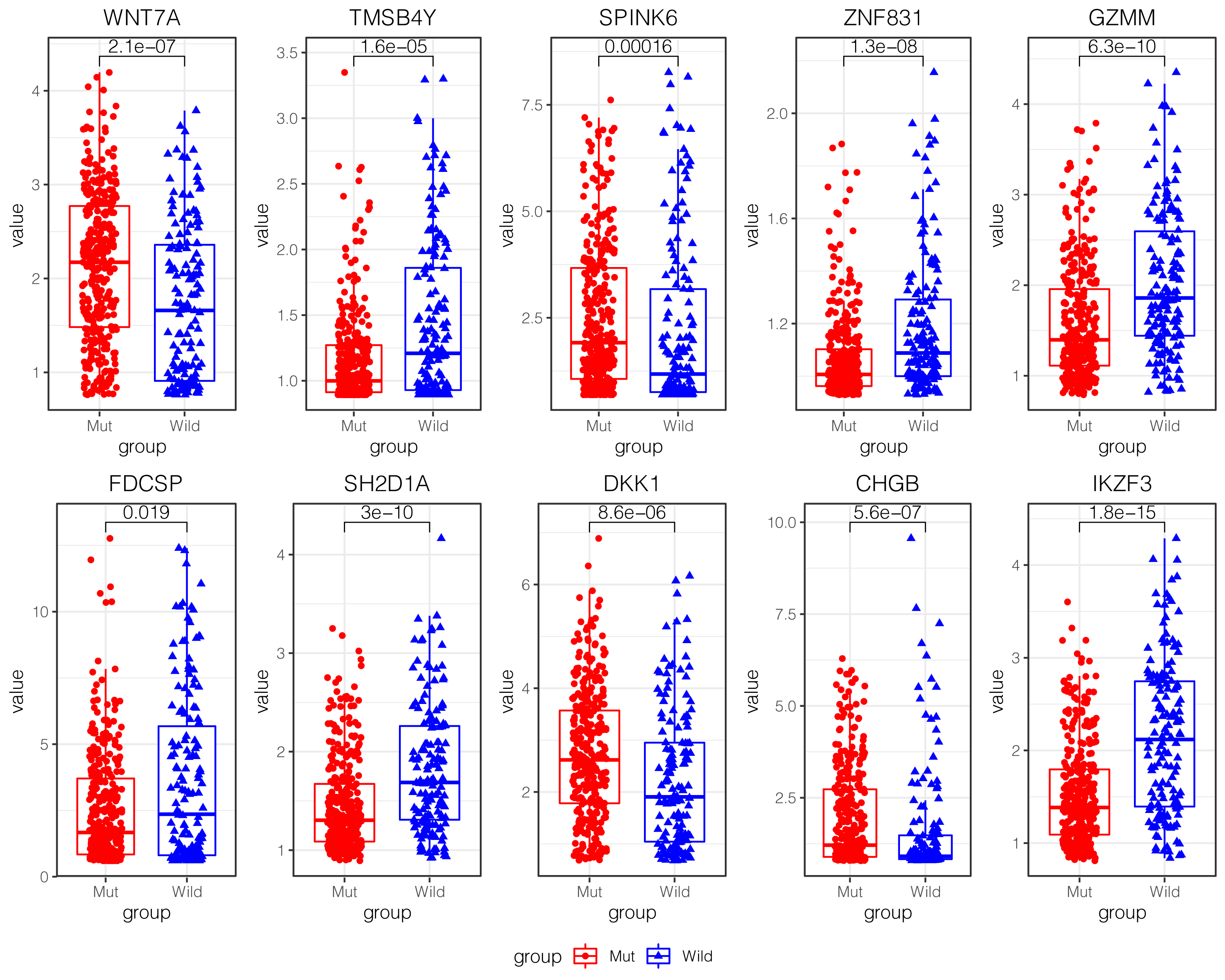

Supplement: Supplementary file 2 — Additional file 2: Supplementary Figure 2. The boxplot of ten prognostic genes expression in model of TCGA HNSC samples of TP53 mutation and wildtype. The p values were calculated by Kruskal-Wallis test. [file 12885_2021_8765_MOESM2_ESM.tif]

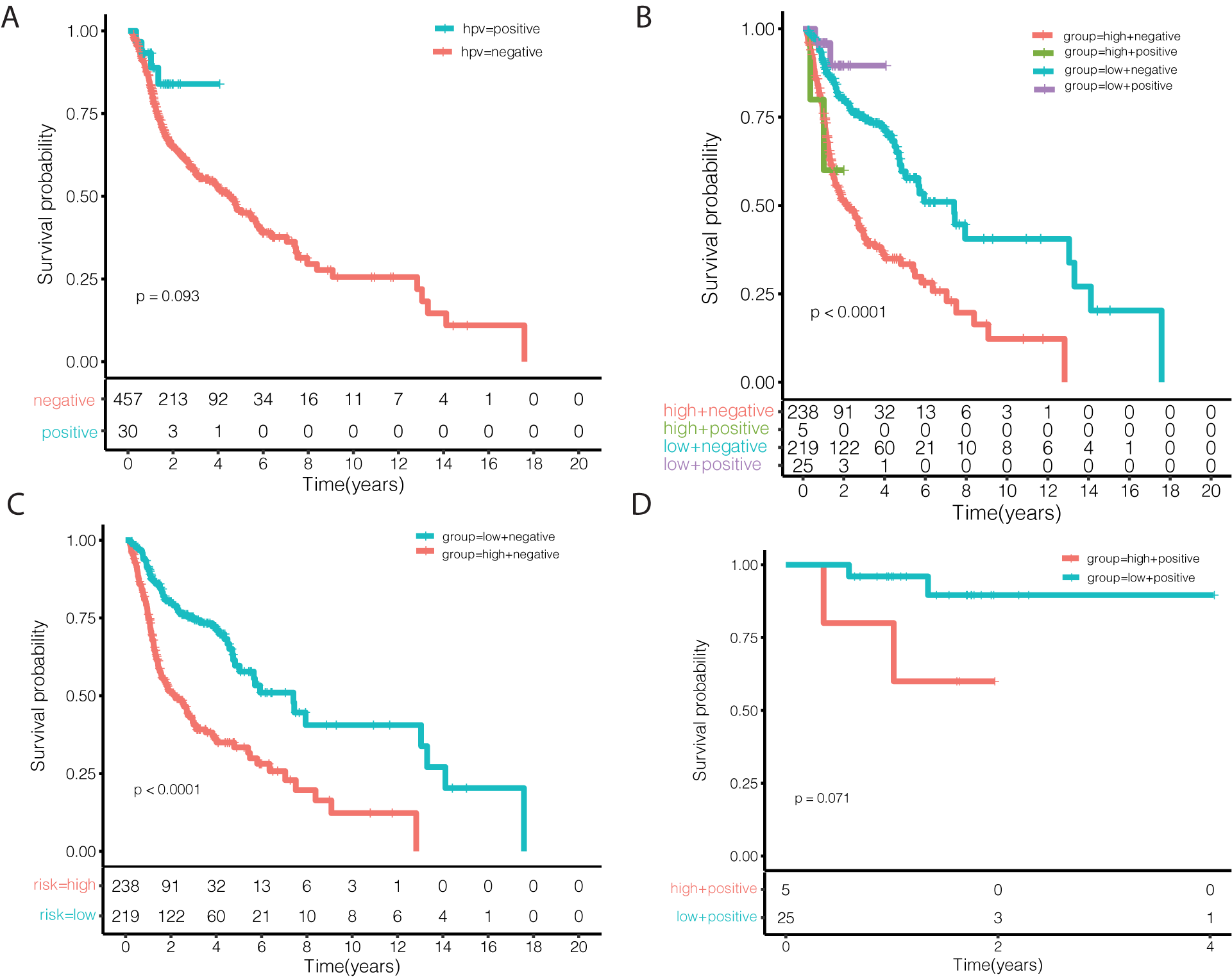

Supplement: Supplementary file 3 — Additional file 3: Supplementary Figure 3. The KM survival plot TCGA HNSC patients stratified by HPV status and risk model. (A) HPV+ vs HPV− group. (B) HPV+ with high−/low-risk group. (C) HPV+ subgroup with high−/low-risk group. (D) HPV− subgroup with high−/low-risk group. [file 12885_2021_8765_MOESM3_ESM.tif]

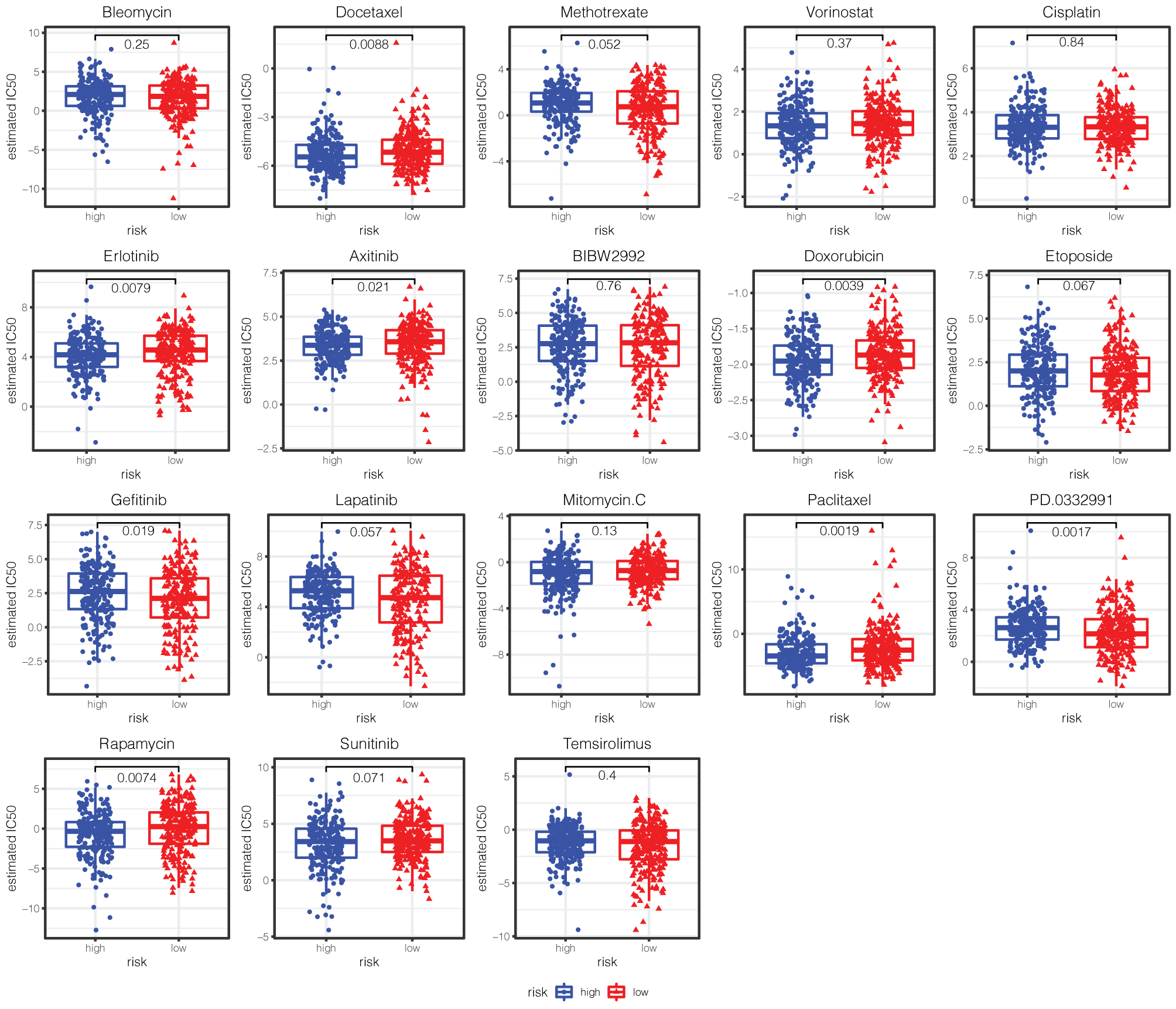

Supplement: Supplementary file 4 — Additional file 4: Supplementary Figure 4. Drugs in clinics or in test for HNSC without significant differential chemotherapeutic responses (p ≥ 0.001) in high- and low-risk patients. The results were predicted by the pRRopheic package in the R language, and the difference of IC50 were assessed with Kruskal-Wallis test. [file 12885_2021_8765_MOESM4_ESM.tif]

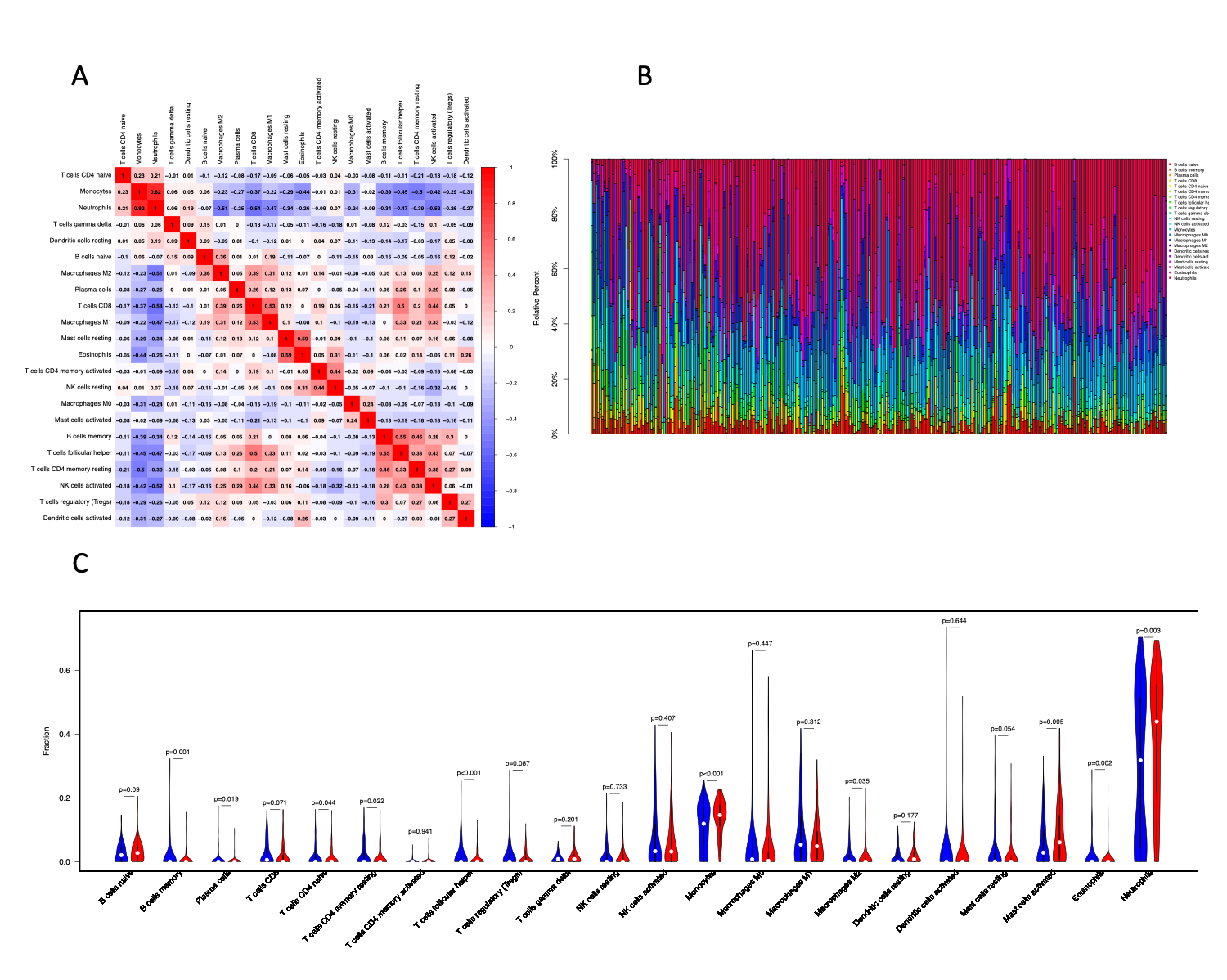

Supplement: Supplementary file 5 — Additional file 5: Supplementary Figure 5. Immune cell infiltration landscapes in high- and low-risk patients with HNSC in GSE65858 cohort. (A) Correlation matrix for immune cells.; (B) Scaled immune cell infiltration proportions in high- and low-risk patients (C) Differences of immune cell infiltrations between high- and low-risk patients. The difference of cell fractions were assessed with Kruskal-Wallis test. [file 12885_2021_8765_MOESM5_ESM.tiff]
