# Supplementary material for: A TP53 mutation model for the prediction of prognosis and therapeutic responses in head and neck squamous cell carcinoma
Source: BMC Cancer. 2021 Sep 16;21:1035. doi: 10.1186/s12885-021-08765-w (PMC8447564; doi:10.1186/s12885-021-08765-w)
Supplement: Supplementary file 6 — Additional file 6: Supplemental Table 1. The immune and clinic characteristics between HPV negative patients and positive patients in TCGA HNSC. Briefly speaking HPV + HNSC has more infiltrated immune cells, especially tumor suppressive cells like memory B cell, CD8 T cells, activated memory CD4 T cells, T cells follicular helper, and less pro-tumor cells like Macrophages M2. Interestingly HPV + HNSC has a greater proportion of low risk group, and those HPV positive patients were younger (median age:57 vs 61) and of high pathological grade. [file 12885_2021_8765_MOESM6_ESM.docx]

Supplemental Table 1. The characteristics between HPV negative patients and positive patients

|  | Negative | Positive | p.overall |
| --- | --- | --- | --- |
|  | *N=457* | *N=30* |  |
| B cells naive | 0.02 [0.00;0.06] | 0.02 [0.01;0.07] | 0.445 |
| B cells memory | 0.00 [0.00;0.00] | 0.00 [0.00;0.00] | 0.038 |
| Plasma cells | 0.01 [0.00;0.06] | 0.04 [0.01;0.07] | 0.077 |
| T cells CD8 | 0.07 [0.03;0.14] | 0.15 [0.11;0.22] | <0.001 |
| T cells CD4 naive | 0.00 [0.00;0.00] | 0.00 [0.00;0.00] | 0.738 |
| T cells CD4 memory resting | 0.12 [0.05;0.18] | 0.07 [0.00;0.12] | 0.005 |
| T cells CD4 memory activated | 0.04 [0.01;0.08] | 0.08 [0.04;0.11] | 0.002 |
| T cells follicular helper | 0.02 [0.00;0.05] | 0.06 [0.04;0.08] | <0.001 |
| T cells regulatory (Tregs) | 0.01 [0.00;0.02] | 0.03 [0.01;0.05] | <0.001 |
| T cells gamma delta | 0.00 [0.00;0.00] | 0.00 [0.00;0.00] | 0.598 |
| NK cells resting | 0.02 [0.00;0.04] | 0.00 [0.00;0.03] | 0.203 |
| NK cells activated | 0.00 [0.00;0.02] | 0.00 [0.00;0.02] | 0.333 |
| Monocytes | 0.00 [0.00;0.00] | 0.00 [0.00;0.01] | 0.021 |
| Macrophages M0 | 0.20 [0.12;0.31] | 0.13 [0.06;0.20] | 0.001 |
| Macrophages M1 | 0.08 [0.04;0.13] | 0.08 [0.06;0.11] | 0.860 |
| Macrophages M2 | 0.09 [0.06;0.13] | 0.06 [0.05;0.09] | 0.004 |
| Dendritic cells resting | 0.03 [0.01;0.06] | 0.02 [0.01;0.04] | 0.411 |
| Dendritic cells activated | 0.02 [0.00;0.05] | 0.00 [0.00;0.03] | 0.118 |
| Mast cells resting | 0.02 [0.00;0.05] | 0.03 [0.00;0.05] | 0.312 |
| Mast cells activated | 0.00 [0.00;0.04] | 0.00 [0.00;0.00] | 0.106 |
| Eosinophils | 0.00 [0.00;0.00] | 0.00 [0.00;0.00] | 0.440 |
| Neutrophils | 0.00 [0.00;0.01] | 0.00 [0.00;0.01] | 0.049 |
| group: |  |  | <0.001 |
| high | 238 (52.1%) | 5 (16.7%) |  |
| low | 219 (47.9%) | 25 (83.3%) |  |
| age | 61.0 [53.0;69.0] | 57.0 [53.0;60.0] | 0.006 |
| gender: |  |  | 0.063 |
| female | 124 (27.1%) | 3 (10.0%) |  |
| male | 333 (72.9%) | 27 (90.0%) |  |
| alcohol: |  |  | 0.362 |
| No | 144 (31.5%) | 6 (20.0%) |  |
| Not Reported | 11 (2.41%) | 0 (0.00%) |  |
| Yes | 302 (66.1%) | 24 (80.0%) |  |
| cStage: |  |  | 1.000 |
| stage i | 24 (5.96%) | 1 (5.88%) |  |
| stage ii | 65 (16.1%) | 3 (17.6%) |  |
| stage iii | 75 (18.6%) | 3 (17.6%) |  |
| stage iv | 239 (59.3%) | 10 (58.8%) |  |
| grade: |  |  | 0.001 |
| G1 | 59 (13.3%) | 0 (0.00%) |  |
| G2 | 277 (62.2%) | 14 (60.9%) |  |
| G3 | 109 (24.5%) | 7 (30.4%) |  |
| G4 | 0 (0.00%) | 2 (8.70%) |  |

Note: The values of continuous variable such as cell content were showed as median [first quantile, third quantile]. Categorical variables were showed in number(percentage), and difference were detected by Chi squared test. Kruskal Wallis test was used to determine statistically significant difference between groups.
