# Supplementary material for: A TP53 mutation model for the prediction of prognosis and therapeutic responses in head and neck squamous cell carcinoma
Source: BMC Cancer. 2021 Sep 16;21:1035. doi: 10.1186/s12885-021-08765-w (PMC8447564; doi:10.1186/s12885-021-08765-w)
Supplement: Supplementary file 7 — Additional file 7: Supplementary Table 2. The immune cell infiltration differences between patients of high risk and low risk stratified by HPV status. The immune cell proportion differences were mainly present in HPV-HNSC patient. Significant p values were marked boldly. [file 12885_2021_8765_MOESM7_ESM.docx]

**Supplemental Table 2 The immune cell infiltration differences between patients of high risk and low risk stratified by HPV status**

|  | Negative | | |  | Positive | | |
| --- | --- | --- | --- | --- | --- | --- | --- |
|  | high | low | p.overall |  | high | low | p.overall |
|  | *N=238* | *N=219* |  |  | *N=5* | *N=25* |  |
| B cells naive | 0.01 [0.00;0.04] | 0.03 [0.00;0.08] | 0.004 |  | 0.00 [0.00;0.03] | 0.02 [0.01;0.07] | 0.155 |
| B cells memory | 0.00 [0.00;0.00] | 0.00 [0.00;0.00] | 0.551 |  | 0.00 [0.00;0.00] | 0.00 [0.00;0.00] | 0.668 |
| Plasma cells | 0.01 [0.00;0.03] | 0.02 [0.00;0.08] | <0.001 |  | 0.02 [0.01;0.03] | 0.04 [0.01;0.08] | 0.133 |
| T cells CD8 | 0.05 [0.02;0.11] | 0.10 [0.05;0.17] | <0.001 |  | 0.14 [0.05;0.18] | 0.15 [0.12;0.22] | 0.487 |
| T cells CD4 naive | 0.00 [0.00;0.00] | 0.00 [0.00;0.00] | 0.873 |  | 0.00 [0.00;0.00] | 0.00 [0.00;0.00] | 0.025 |
| T cells CD4 memory resting | 0.12 [0.07;0.18] | 0.11 [0.04;0.17] | 0.076 |  | 0.15 [0.00;0.15] | 0.07 [0.00;0.11] | 0.634 |
| T cells CD4 memory activated | 0.03 [0.00;0.07] | 0.05 [0.02;0.09] | <0.001 |  | 0.10 [0.08;0.13] | 0.07 [0.04;0.11] | 0.278 |
| T cells follicular helper | 0.01 [0.00;0.04] | 0.03 [0.01;0.05] | <0.001 |  | 0.07 [0.05;0.07] | 0.06 [0.03;0.08] | 0.978 |
| T cells regulatory (Tregs) | 0.00 [0.00;0.02] | 0.01 [0.00;0.03] | <0.001 |  | 0.00 [0.00;0.01] | 0.04 [0.02;0.06] | 0.061 |
| T cells gamma delta | 0.00 [0.00;0.00] | 0.00 [0.00;0.00] | 0.207 |  | 0.00 [0.00;0.00] | 0.00 [0.00;0.00] | 0.520 |
| NK cells resting | 0.02 [0.00;0.04] | 0.02 [0.00;0.04] | 0.499 |  | 0.06 [0.03;0.06] | 0.00 [0.00;0.03] | 0.009 |
| NK cells activated | 0.00 [0.00;0.03] | 0.00 [0.00;0.02] | 0.495 |  | 0.00 [0.00;0.00] | 0.00 [0.00;0.02] | 0.385 |
| Monocytes | 0.00 [0.00;0.00] | 0.00 [0.00;0.00] | 0.151 |  | 0.01 [0.00;0.01] | 0.00 [0.00;0.00] | 0.017 |
| Macrophages M0 | 0.23 [0.15;0.35] | 0.16 [0.09;0.26] | <0.001 |  | 0.08 [0.04;0.20] | 0.15 [0.06;0.17] | 0.636 |
| Macrophages M1 | 0.09 [0.03;0.14] | 0.08 [0.05;0.12] | 0.558 |  | 0.11 [0.00;0.11] | 0.08 [0.06;0.11] | 0.933 |
| Macrophages M2 | 0.10 [0.06;0.14] | 0.09 [0.06;0.12] | 0.012 |  | 0.08 [0.06;0.08] | 0.06 [0.05;0.10] | 0.303 |
| Dendritic cells resting | 0.02 [0.00;0.06] | 0.03 [0.01;0.06] | 0.074 |  | 0.01 [0.01;0.04] | 0.02 [0.01;0.04] | 0.559 |
| Dendritic cells activated | 0.02 [0.00;0.06] | 0.02 [0.00;0.04] | 0.577 |  | 0.03 [0.00;0.12] | 0.00 [0.00;0.03] | 0.220 |
| Mast cells resting | 0.01 [0.00;0.04] | 0.02 [0.00;0.05] | 0.335 |  | 0.00 [0.00;0.00] | 0.03 [0.01;0.05] | 0.040 |
| Mast cells activated | 0.00 [0.00;0.05] | 0.00 [0.00;0.03] | 0.118 |  | 0.02 [0.00;0.15] | 0.00 [0.00;0.00] | 0.042 |
| Eosinophils | 0.00 [0.00;0.00] | 0.00 [0.00;0.00] | 0.040 |  | 0.00 [0.00;0.00] | 0.00 [0.00;0.00] | 0.184 |
| Neutrophils | 0.00 [0.00;0.01] | 0.00 [0.00;0.01] | 0.008 |  | 0.01 [0.00;0.01] | 0.00 [0.00;0.00] | 0.415 |

Note: The values of continuous variable such as cell content were showed as median[first quantile, third quantile]. Kruskal Wallis test was used to determine statistically significant difference between groups.
