# Supplementary material for: A TP53 mutation model for the prediction of prognosis and therapeutic responses in head and neck squamous cell carcinoma
Source: BMC Cancer. 2021 Sep 16;21:1035. doi: 10.1186/s12885-021-08765-w (PMC8447564; doi:10.1186/s12885-021-08765-w)
Supplement: Supplementary file 8 — Additional file 8: Supplementary Table 3. Univariate Cox analysis results of the 30 significant genes. Ten genes in risk model were marked boldly. Padj value were calculated by Bonferroni correction. [file 12885_2021_8765_MOESM8_ESM.docx]

Supplementary Table 3 univariate Cox analysis results of the 30 significant genes

| Gene | HR | HR.95L | HR.95H | pvalue | padj |
| --- | --- | --- | --- | --- | --- |
| *POPDC3* | 1.27842947 | 1.10565569 | 1.4782015 | 0.00091388 | 0.00726424 |
| *WNT7A** | 1.41207214 | 1.1970718 | 1.66568766 | 4.23E-05 | 0.00224391 |
| *IGF2BP2* | 1.29258765 | 1.1122886 | 1.5021127 | 0.00081282 | 0.00680199 |
| *TMSB4Y** | 0.55674067 | 0.39009892 | 0.79456814 | 0.00125067 | 0.00781573 |
| *SPINK6** | 0.87101293 | 0.80123084 | 0.94687258 | 0.00119014 | 0.00781573 |
| *UBASH3A* | 0.53069893 | 0.36946968 | 0.76228544 | 0.00060566 | 0.00618424 |
| *CD40LG* | 0.37448219 | 0.21528273 | 0.65140808 | 0.00050617 | 0.00618424 |
| *ZNF831** | 0.19660723 | 0.08409212 | 0.4596674 | 0.00017426 | 0.004618 |
| *SIRPG* | 0.67483819 | 0.53688146 | 0.84824419 | 0.00075031 | 0.00662775 |
| *GPR18* | 0.42238284 | 0.24912236 | 0.71614312 | 0.00137713 | 0.00781573 |
| *LHX1* | 1.65576608 | 1.21609589 | 2.25439566 | 0.00136257 | 0.00781573 |
| *SYCP2* | 0.72366393 | 0.59164375 | 0.88514325 | 0.00164867 | 0.00873797 |
| *CXCR3* | 0.73043445 | 0.60619354 | 0.88013886 | 0.00095943 | 0.00726424 |
| *GZMM** | 0.64309833 | 0.51803353 | 0.79835655 | 6.31E-05 | 0.00250753 |
| *FDCSP** | 0.89686563 | 0.8441284 | 0.95289765 | 0.00043092 | 0.00618424 |
| *INPP5D* | 0.67206532 | 0.54167444 | 0.83384367 | 0.00030485 | 0.00538566 |
| *SERPINE1* | 1.18599209 | 1.06796552 | 1.31706241 | 0.00142551 | 0.00781573 |
| *SH2D1A** | 0.64611077 | 0.49502922 | 0.84330198 | 0.00130867 | 0.00781573 |
| *DKK1** | 1.25625716 | 1.13785896 | 1.3869751 | 6.27E-06 | 0.00099684 |
| *F2RL1* | 1.38145743 | 1.16132886 | 1.64331112 | 0.00026345 | 0.00538566 |
| *INHBA* | 1.21219593 | 1.08604315 | 1.35300239 | 0.00059892 | 0.00618424 |
| *CHGB** | 1.19124312 | 1.09138933 | 1.30023278 | 8.94E-05 | 0.0028415 |
| *ANO1* | 1.17452636 | 1.06505735 | 1.29524684 | 0.00127023 | 0.00781573 |
| *SHANK2* | 1.37707264 | 1.13178575 | 1.67551947 | 0.00138924 | 0.00781573 |
| *PRSS12* | 0.71099744 | 0.60623754 | 0.83386019 | 2.74E-05 | 0.00217853 |
| *ARHGAP4* | 0.75984007 | 0.64923112 | 0.88929337 | 0.00062231 | 0.00618424 |
| *SRPX* | 1.2649757 | 1.11458277 | 1.43566145 | 0.00027289 | 0.00538566 |
| *IKZF3** | 0.69586505 | 0.56608019 | 0.85540561 | 0.00057553 | 0.00618424 |
| *MS4A1* | 0.63035176 | 0.48439339 | 0.82029061 | 0.0005945 | 0.00618424 |
| *CD19* | 0.59884134 | 0.44494303 | 0.80597049 | 0.0007164 | 0.00662775 |

Note: Genes labeled with “*” were included in the risk model.
