# Supplementary material for: A TP53 mutation model for the prediction of prognosis and therapeutic responses in head and neck squamous cell carcinoma
Source: BMC Cancer. 2021 Sep 16;21:1035. doi: 10.1186/s12885-021-08765-w (PMC8447564; doi:10.1186/s12885-021-08765-w)
Supplement: Supplementary file 9 — Additional file 9: Supplementary Table 4. Information of the ten genes in the prognostic model. Most of the ten genes were correlated with tumor or immune functions. Padj value and HR from coxph analysis and coefficients from risk model formula were displayed in the table. [file 12885_2021_8765_MOESM9_ESM.docx]

Supplementary Table 4 Information of the genes in the prognostic model.

| gene name | padj | HR | coefficient | discription |
| --- | --- | --- | --- | --- |
| *WNT7A* | 0.00224391 | 1.41207214 | 0.27508625 | This gene is a member of the WNT gene family, promotes cancer invasion through the WNT/β-catenin pathway and correlated with worse prognosis. |
| *TMSB4Y* | 0.00781573 | 0.55674067 | -0.5406673 | This gene is a candidate tumor suppressor. |
| *SPINK6* | 0.00781573 | 0.87101293 | -0.1956309 | This role of this gene in cancer is ambivalent in various cancers and papers. According to reports, it inhibits tumorigenesis in HCC ^[1],^ and promotes metastasis by activating EGFR in nasopharyngeal carcinoma ^[2]^. |
| *ZNF831* | 0.004618 | 0.19660723 | -3.1999347 | Previous understanding of this gene focuses on cardiovascular disease and infections, especially HIV. The part it taken in cancer is unclear yet. |
| *GZMM* | 0.00250753 | 0.64309833 | -0.4169944 | This gene codes a protein products belong to granzymes, a distinct subset of neutral serine proteases. It is a immune effector molecules in large cytoplasmic granules stored in human NKs and activated lymphocytes. |
| *FDCSP* | 0.00618424 | 0.89686563 | -0.0579227 | This gene encodes a small secreted protein that is expressed in follicular dendritic cells. This protein specifically binds to activated B cells, and functions as a regulator of antibody responses. It is also thought to contribute to tumor metastases by promoting cancer cell migration and invasion. |
| *SH2D1A* | 0.00781573 | 0.64611077 | 0.58426326 | This gene encodes a protein that plays a major role in the bidirectional stimulation of T and B cells. This protein contains an SH2 domain and a short tail. This protein can also bind to other related surface molecules that are expressed on activated T, B and NK cells, thereby modifying signal transduction pathways in these cells. |
| *DKK1* | 0.00099684 | 1.25625716 | 0.10207575 | A member of the dickkopf family of proteins. The encoded protein binds to the LRP6 co-receptor and inhibits beta-catenin-dependent Wnt signaling. Elevated expression of this gene has been observed in numerous human cancers and this protein may promote proliferation, invasion and growth in cancer cell lines. |
| *CHGB* | 0.0028415 | 1.19124312 | 0.13111763 | This gene encodes a tyrosine-sulfated secretory protein abundant in peptidergic endocrine cells and neurons. Its evaluated expression in serum and tissues of breast cancer patients was reported. |
| *IKZF3* | 0.00618424 | 0.69586505 | 0.62912215 | This gene encodes a member of the Ikaros family of zinc-finger proteins. Three members of this protein family (Ikaros, Aiolos and Helios) are hematopoietic-specific transcription factors involved in the regulation of lymphocyte development. This gene product is a transcription factor that is important in the regulation of B lymphocyte proliferation and differentiation. |

Note:

[1] Ge, K. , Huang, J. , Wang, W. , Gu, M. , Dai, X. , Xu, Y. , Wu, H. , Li, G. , Lu, H. , Zhong, J. , & Huang, Q. . (2017). Serine protease inhibitor kazal-type 6 inhibits tumorigenesis of human hepatocellular carcinoma cells via its extracellular action.. 8(4), 5965-5975.

[2] Zheng, L. , Yang, J. , Cao, Y. , Peng, L. , Sun, R. , Xie, P. , Wang, M. , Meng, D. , Luo, D. , Zou, X. , Chen, M. , Mai, H. , Guo, L. , Guo, X. , Shao, J. , Huang, B. , Zhang, W. , & Qian, C. . (2017). SPINK6 Promotes Metastasis of Nasopharyngeal Carcinoma via Binding and Activation of Epithelial Growth Factor Receptor.. 77(2), 579-589.
